# Development of A 3D Tissue Slice Culture Model for the Study of Human Endometrial Repair and Regeneration

**DOI:** 10.3390/biom10010136

**Published:** 2020-01-14

**Authors:** Shanmugam Muruganandan, Xiujun Fan, Sabita Dhal, Nihar R. Nayak

**Affiliations:** 1 Perinatal Research Initiative, Department of Obstetrics and Gynecology, Wayne State University School of Medicine, Detroit, MI 48201, USA; muruganandan_shanmugam@hsdm.harvard.edu (S.M.); sdhal@med.wayne.edu (S.D.); nnayak@med.wayne.edu (N.R.N.); 2 Department of Developmental Biology, Harvard School of Dental Medicine, 188 Longwood Avenue, Harvard University, Boston, MA 02115, USA; 3 Laboratory of Reproductive Health, Shenzhen Institute of Advanced Technology, Chinese Academy of Sciences, Shenzhen 518055, China

**Keywords:** remodeling of the human endometrium, 3D slice culture system, stem cells

## Abstract

The human endometrium undergoes sequential phases of shedding of the upper functionalis zone during menstruation, followed by regeneration of the functionalis zone from the remaining basalis zone cells, and secretory differentiation under the influence of the ovarian steroid hormones estradiol (E2) and progesterone (P4). This massive tissue regeneration after menstruation is believed to arise from endometrial stromal and epithelial stem cells residing in the basal layer of the endometrium. Although many endometrial pathologies are thought to be associated with defects in these stem cells, studies on their identification and regulation are limited, primarily due to lack of easily accessible animal models, as these processes are unique to primates. Here we describe a robust new method to study endometrial regeneration and differentiation processes using human endometrial tissue slice cultures incorporating an air-liquid interface into a 3D matrix scaffold of type I collagen gel, allowing sustained tissue viability over three weeks. The 3D collagen gel-embedded endometrial tissue slices in a double-dish culture system responded to ovarian steroid hormones, mimicking the endometrial changes that occur in vivo during the menstrual cycle. These changes included the E2-induced upregulation of Ki-67, estrogen receptor (ER), and progesterone receptor (PR) in all endometrial compartments and were markedly suppressed by both P4 and E2 plus P4 treatments. There were also distinct changes in endometrial morphology after E2 and P4 treatments, including subnuclear vacuolation and luminal secretions in glands as well as decidualization of stromal cells, typical characteristics of a progestational endometrium in vivo. This long-term slice culture method provides a unique in vivo-like microenvironment for the study of human endometrial functions and remodeling during early pregnancy and experiments on stem cell populations involved in endometrial regeneration and remodeling. Furthermore, this model has the potential to enable studies on several endometrial diseases, including endometrial cancers and pregnancy complications associated with defects in endometrial remodeling.

## 1. Introduction

An efficient endometrial remodeling is critical for regular cycles of tissue shedding and repair during menstruation and for the preparation of uterus for embryo implantation and pregnancy [1,2]. Ovarian hormones estradiol (E2) and progesterone (P4) are known to guide each stage of the tissue remodeling process that ultimately result into the cyclical changes in endometrium [3]. Similarly, the endometrial changes in early pregnancy are also induced by actions of P4 on a variety of cell types including the endometrial stromal cells that undergo the characteristic decidualization reaction essential for a successful implantation [4]. This decidualization of the endometrium begins at the mid-secretory phase of the menstrual cycle that do not require the presence of fertilized conceptus but can be maintained only when pregnancy occurs with P4 remaining high [4]. Under the influence of P4, the endometrial stromal cells differentiate into the decidual cells acquiring unique phenotype with several biological functions such as the modulation of expression and function of growth factors, tissue factor (TF), type-1 plasminogen activator inhibitor (PAI-1), plasminogen activators (PAs), matrix remodeling proteins (e.g., matrix metalloproteinases (MMPs), tissue inhibitor of matrix metalloproteinases (TIMPs)) and signaling molecules (e.g., connexin 43 and endothelin-1 (ET-1)) that modify extracellular matrix (ECM) turnover and prepare the uterus for implantation [5,6]. After implantation, the trophoblast remodeling of maternal uterine spiral arteries is critical to adequate placental perfusion for establishing a successful pregnancy [2]. Deep trophoblast invasion, a key cellular process in spiral artery remodeling is dynamically regulated by extravillous trophoblast (EVT) differentiation of villous cytotrophoblasts (VCTs) that confers invasive capacity to the cells [7]. Concerted actions of several signaling molecules derived from trophoblasts and decidual cells coordinate the ordered differentiation and function of trophoblasts required for promoting maternal vascular adaptations to pregnancy [7,8]. These functions of decidua are necessary to prevent hemorrhage during vascular remodeling and EVT invasion [9]. On the other hand, in non-fertile cycles, P4 withdrawal in decidual cells induces cell death and dedifferentiation that reduces tissue factor and PAI-1 while increasing PA, MMPs and ET-1 causing ECM degradation, fibrinolysis, endometrial breakdown, shedding and menstrual-associated bleeding [1,10,11].

Any derangement in the remodeling process can result into abnormal conditions causing infertility, pathological endometrial bleeding disorders, endometriosis, endometrial hyperplasia and endometrial cancer [12,13]. Although the physiological and pathological changes in the endometrium during remodeling are well characterized, the cellular mechanisms that drive the programmed events of the endometrial tissue remodeling are still not completely understood. Recent studies provide evidence for endometrial stem cell (ESC) populations that contribute to endometrial regeneration and repair [14,15]. However, these studies have either used rodent models that does not experience menstrual shedding or artificial mouse models with administration and withdrawal of exogenous progesterone rather than the naturally menstruating women with declining hormone levels caused by the demise of corpus luteum. The recently discovered, naturally menstruating spiny mouse, however, has the scope for being developed as an attractive rodent model, but is still limited by the milder degree of remodeling in the luminal epithelium, as well as a lack of remodeling in the glands that can cause variations from primates [16]. Overall, the variations in rodent models can cause varied onset of bleeding, may involve different cell types and initiate artificially-induced remodeling process than the endogenously controlled remodeling program in a naturally menstruating women. Thus conventional approaches such as genetic lineage tracing or fate mapping experiments to identify stem cells involved in endometrial remodeling during menstrual cycle are limited by lack of laboratory species that naturally menstruate.

Recently, the development of 3D organoid cultures has gained attention as a powerful tool to study endometrial remodeling [17,18,19,20,21,22,23,24]. These cultures exploit the self-organization ability of endometrial cell types that reproduce many aspects of the phenotypical characteristics of endometrium [17,18,19,20,21,22,23,24]. The flexibility, reliability and reproducibility of organoid cultures derived from human endometrial cell types have largely helped to overcome the obstacle due to lack of animal models. Apart from the phenotypes that can be modeled by 3D reconstructions of endometrial epithelium or stromal cell compartments and their interactions, primate endometrium is also reported to exhibit quadripartite horizontal zonation consisting of functionalis I (luminal epithelium), functionalis II (upper straight gland segments), basalis III (middle gland segments) and basalis IV (bottoms of the glands),each of which have a distinctive stroma and microvasculature that constantly change during the cycle [25]. The different endometrial zones offer distinct microenvironments and it has been shown that some of the key cellular functions such as proliferative responses during E2 dominance and decidualization reaction could vary depending on the zones to which the cells are housed [25]. Likewise, the vascular changes leading to menstruation and the selective infiltration of certain leukocyte subsets are unique to the endometrial zonation in primates [26]. 

To establish an in vitro system that can authentically recapitulate the zone-specific endometrial changes occurring in menstruating women, we developed a 3D tissue-slice culture model that preserved viability of endometrial cells in the tissue for periods over 3 weeks. Characterization of the hormonal response profiles in the 3D culture model recapitulated several aspects of the endometrial changes that occur during menstrual cycle suggesting that these models can provide invaluable tools for translational research in the study of stem cell populations involved in endometrial regeneration and repair. 

## 2. Materials and Methods

### 2.1. Endometrial Tissue Collection

**Figure 1:**
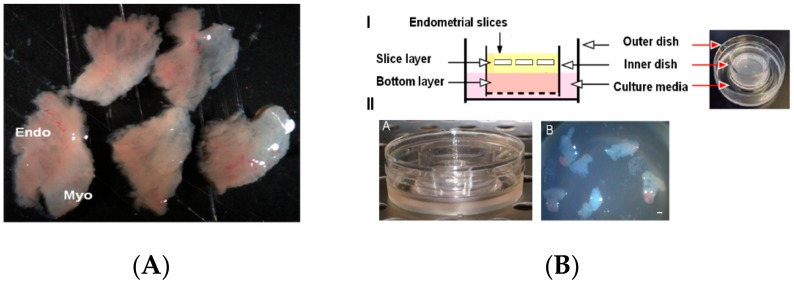
Artificial 3D cell culture model system for endometrial slice culture. (**A**) Slices from full thickness human endometrium. (**B**) I- Schematic representation of double-dish model system for ex vivo culturing of endometrial slices. (**B**) II- Representative images showing endometrial tissue slices grafted onto the surface of a 30-mm inner dish in Cellmatrix Type I-A, Nitta Gelatin with the help of a hydrophilic polytetrafluoroethylene membrane while the 60-mm outer dish contains the 3D cell culture medium. Myo, myometrium; Endo, endometrium. Scale bar = 1 mm.

Women with regular cycles (25–33 days) undergoing hysterectomy for benign indications during the proliferative phase were enrolled in this study, as described previously [27,28]. Written informed consent was obtained from all participants with approved protocols from the Stanford University Committee on the Use of Human Subjects in Medical Research. At least 9 samples from different human subjects were used and samples from subjects with fibroids or benign polyps were excluded from this study. Full thickness endometrial tissue samples were obtained that included superficial and basal endometrium plus the endometrial-myometrial junction. Then, the samples were sliced into 2-mm thick slices (Figure 1A), washed in PBS, and embedded in a three-dimensional (3D) collagen gel using a double-dish culture system [29,30].

### 2.2. Three-Dimensional (3D) Human Tissue Slice Culture System

The human endometrial tissue slices were cultured using a double dish culture system as previously described [29,30]. We used optimized culture conditions by embedding the human endometrial tissue slices in a collagen gel to preserve the slice morphology and endometrial zonation with functions for long-term studies on stem cells and tissue remodeling. The collagen gel enabled the slices to be held in place while preserving the specificity of cell types housed in all endometrial zones. Briefly, 5–6 endometrial slices embedded in 1 mL of collagen gel (Cellmatrix Type I-A, Nitta Gelatin) were poured into a 30-mm inner dish (Millicell-CM, Millipore, Burlington, VT, USA) (Figure 1B) with a hydrophilic polytetrafluoroethylene membrane at the bottom to form an acellular layer. Then, the inner dish was placed into a 60-mm outer dish containing 1.5 mL 3D cell culture medium (Ham’s F12 containing 20% fetal bovine serum, 100 U/mL penicillin, and 100 µg/mL streptomycin, and the culture assembly was incubated at 37 °C in a fully humidified atmosphere of 5% CO_2_ in air at 37 °C. After 2 days, the 3D cell culture medium was changed and tissue slices in different dishes were subjected to various hormonal treatments (E2 — 10 nmol/L, P4 — 100 nmol/L). The tissue slices were cultured for 21 days and examined in different functional experiments to assess feasibility of transduction and hormonal treatment. Subsequent to hormonal treatments, the slices were processed for histology and immunohistochemistry following a standard protocol.

### 2.3. Adenovirus-Mediated Gene Delivery

The human endometrial tissue slices cultured ex vivo for a 3-week period were assessed for their suitability for developing endometrial gene therapy protocols by testing the ex vivo β-galactosidase gene delivery efficiency using adenoviral vectors. Replication-defective adenoviral vectors expressing lacZ (Ad5CMVntLacZ, AdLacZ) were obtained from the Gene Transfer Vector Core at the University of Iowa (Iowa City). The slices were treated with AdLacZ (1 × 10^8^ PFU) 3 days before the end of the 21-day culture period and stained by the β-galactosidase staining procedure (LacZ staining) after the end of 72 h transduction period that coincided with the end of 21 day culture period.

### 2.4. LacZ Staining

To examine the expression of LacZ, the AdLacZ-treated 3D endometrial tissue slice cultures were processed for lacZ staining following a standard protocol as described before [31]. Briefly, the tissue slices were fixed at 4 °C for 30 min using 4% PFA in PBS (pH 7.2) followed by washing at room temperature for 15 min with a wash buffer containing phosphate buffer saline (PBS) in the presence of 2 mM MgCl2, 0.01% deoxycholate, and 0.02% NP-40. The tissue slices were subsequently stained at 37 °C for 16 h with 5-bromo-4-chloro-3-indolyl-beta-Dgalactopyranoside (X-gal) staining solution that was prepared by dissolving X-Gal (1 mg/mL), K3Fe(CN)6 (5 mM), and K4Fe(CN)6 (5 mM) in the wash buffer. The stained tissue slices were washed in PBS and examined for the presence of LacZ staining.

### 2.5. Histology and Immunohistochemistry

Immunohistochemistry (IHC) was performed in the endometrial slices after fixation with 4% paraformaldehyde (PFA) in phosphate buffer saline (PBS) according to our previously published methods [31,32]. Briefly, 7-µm thick cryostat sections were mounted directly on Superfrost Plus slides (Fisher Scientific, USA). The sections were fixed in 0.2% picric acid and 2% paraformaldehyde in phosphate buffered saline for 10 in at room temperature. The slides were treated twice for 2 min each with solutions that contained 1.5% polyvinylpyrollidone (PVP) in 85% ethanol, twice for 7 min each in 1.5% polyvinylpyrollidone (PVP) + 0.37% glycine in PBS, and in 1.5% polyvinylpyrollidone (PVP) + 0.1% gelatin in PBS at 4 °C. Endogenous peroxidases were inhibited by incubating the slides for 45 min in a solution containing glucose oxidase (1 unit/mL), sodium azide (1 mM) and glucose (10 mM) in PBS. Subsequently, the sections were incubated with blocking serum for 20 min, followed by incubation overnight at 4 °C with primary antibodies anti-ER (1D-5; Biogenex, San Ramon, CA, USA), anti-PR (JZB-39; courtesy of Geoffrey Greene, University of Chicago), anti-IGFBP1 (DSL-R00335, Diagnostic Systems Labs., Webster, TX, USA) and anti-Ki-67 (Dako Corp., Carpinteria, CA, USA) that are commercially available and extensively validated by our laboratory and other laboratories for IHC studies in the human endometrium [27,32,33,34,35]. Primary antibodies were detected using biotinylated secondary anti-mouse (for anti-ER, anti-Ki-67) and anti-rat (for anti-PR) IgG that was visualized using the Vectastain kit with fluorescein or Texas Red conjugated avidin (Vector Laboratories, Burlingame, CA, USA). Sections were mounted using Vectastain mounting medium containing nuclear stain DAPI (Vector Laboratories). Images were captured using a Zeiss Axioskop 2 microscope equipped with a Zeiss AxioCam camera system (Carl Zeiss, Jena, Germany).

## 3. Results and Discussion

### 3.1. Establishment of Endometrial Tissue Air-Liquid Interface 3D Cultures

Endometrial biopsy is generally employed for histological assessment as a diagnostic tool for evaluating potential endometrial diseases such as luteal phase defects, endometrial hyperplasia, and endometrial cancer [36,37]. However, evidences from the literature suggests that the cycle to cycle changes, menstrual irregularity in perimenopausal women, inter and intra-observer variability in histological evaluations and poor reproducibility can limit the value causing differences in opinions regarding the use of endometrial biopsy [38,39,40,41]. Likewise, the rapid and effective diagnosis of endometriosis is also limited due to clinically unavailable biomarkers that would sensitively and specifically detect the disease state [42]. Moreover, the physiological events of endometrial remodeling such as endometrial stromal cell (ESC) differentiation, decidualization reaction and the trophoblast remodeling of uterine spiral arteries show striking differences from experimental animal models [43,44,45,46,47,48,49,50] that refute the value of animal models to study human endometrium. Therefore, realistically viable cell culture models are required for understanding the pathology and as well as for developing new drugs to target these diseases. However, the two-dimensional (2D) cultures do not provide more physiologically relevant information in the context of complex interactions between the various cells housed in the different zones of the endometrium [48]. Thus, adapting strategies to culture whole tissue slices containing all the constituent cell types residing within the native tissue environment in their original configuration are required to investigate pathophysiological events of the human endometrium. In an attempt to develop a physiologically relevant model system to mimic the human menstrual cycle under an experimental setting we developed a 3D endometrial tissue slice culture system with samples from full thickness biopsy of human endometrium and used E2 and P4 hormone regimen for stimulating endometrial remodeling. Slices from 5–6 endometrial tissue specimens isolated from full thickness endometrium as shown in Figure 1 were used to establish the 3D air-liquid interface cultures. The endometrial slices were embedded in 1 mL of collagen gel constructed on an acellular bottom layer of collagen placed into 3D culture medium with the help of a double dish assembly.

**Figure 2:**
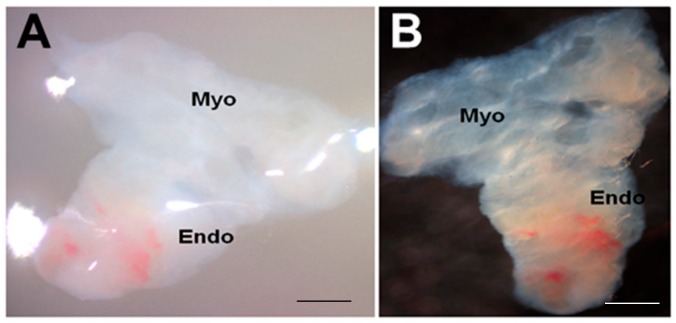
Appearance of endometrial tissue slices after 21 days in culture. (**A**) a tissue slice photographed within the gel; (**B**) the same tissue slice washed in PBS before further processing. Myo, myometrium; Endo, endometrium. Scale bar = 1 mm.

Although further viability studies are needed at 21 days and beyond, the endometrial slices appeared to be viable at the end of 21 days in this 3D-endometrial double dish culture system (Figure 2).

**Figure 3:**
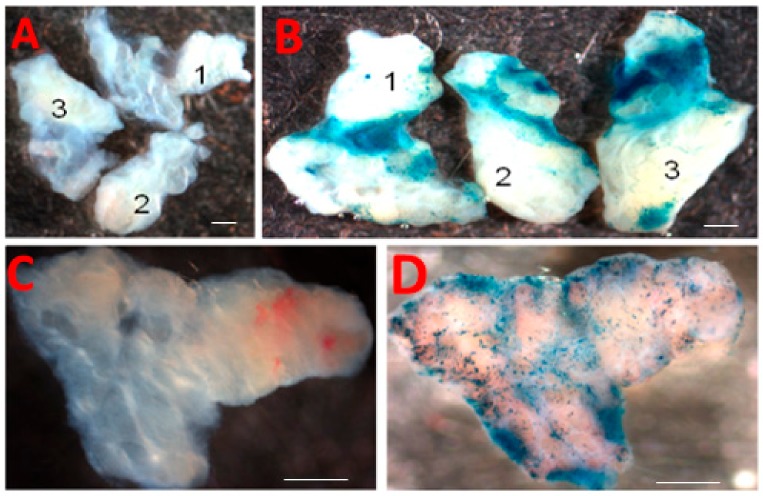
Test for feasibility of functional studies with the 3D endometrial slice culture model. Endometrial slices from 3D model were transduced with adenovirus expressing LacZ (Ad-LacZ) and subsequently stained by the β-galactosidase staining procedure (LacZ staining). (**A**) Tissue slices photographed before LacZ staining; (**B**) the same tissue slices photographed after LacZ staining. (**C**) Magnified view of a tissue slice before LacZ staining; (**D**) Magnified view of the same tissue slice photographed after LacZ staining. Scale bar = 1 mm.

To test the feasibility of functional studies using the 3D model system, we assessed the ability of expression vectors to manipulate gene expression in the slice culture. Same tissue slice that was shown in viability analysis was also examined for its ability to be transduced by adenoviral vectors in the culture system. As shown in Figure 3, transduction of the 3D culture system with an adenoviral vector expressing LacZ allowed for the stable expression of high levels of LacZ as assessed by the β-galactosidase staining procedure. 

Although further studies are essential to determine the specific cell types, the widespread transduction of the lacz gene into the endometrial slices was achieved, suggesting feasibility of functional studies in this model system using adenovirus-mediated gene delivery systems. 

The selection of full thickness endometrium for culture allows the epithelial and stromal cell compartments maintained in the 3D model to study stromal-epithelial interactions during menstrual cycle. The progenitor and stem cells that are housed in the stromal compartment offers the opportunity to further investigate their recruitment and differentiation in the 3D model system. Furthermore, because all the endometrial zones are known to be fully developed in the proliferative phase and the cells in different endometrial zones respond differentially to P4 exposure in vivo during the secretory phase of the menstrual cycle, we used endometrial samples from the proliferative phase in order to validate the ability of this model for normal in vivo-like endometrial zone-specific secretory transformation in response to E2 + P4 treatments. Consistent with the findings from in vivo studies, our results show distinct endometrial zone-specific changes in response to ovarian steroid hormones in this model as described below. However, future studies with endometrial samples from the secretory phase should be examined for the potential of this model for use in studies on implantation and early pregnancy. 

### 3.2. Evaluation of E2/P4 Responses in Endometrial Tissue Air-Liquid Interface 3D Cultures

**Figure 4:**
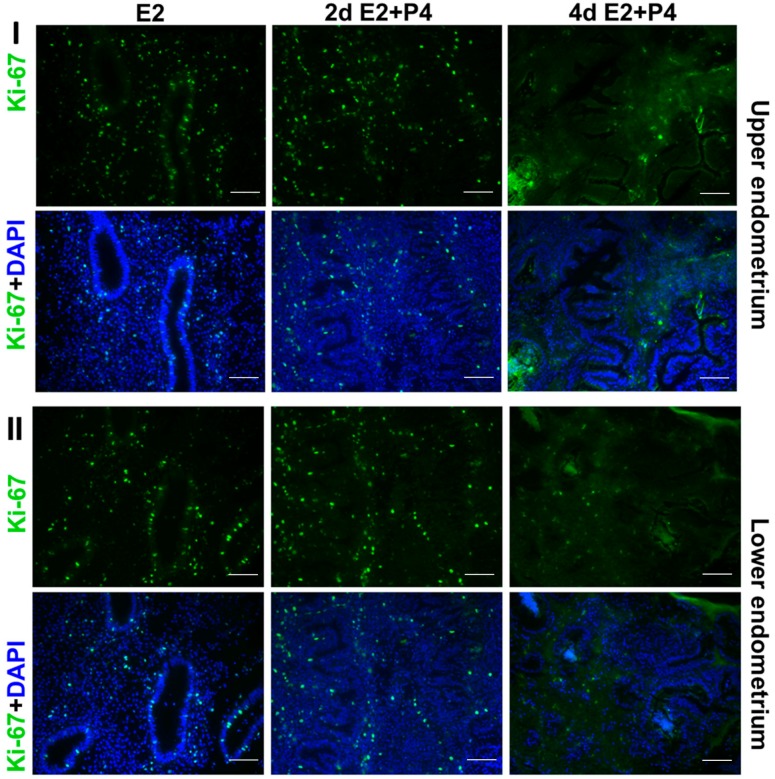
Ki-67 expression (proliferation marker) in the upper (I) and lower (II) zones of full thickness endometrial slices in culture. Four days of P4 alone (data not shown) or E + P4 treatments markedly suppress Ki-67 expression. Scale bar 200 µm; Original magnification 160×.

**Figure 5:**
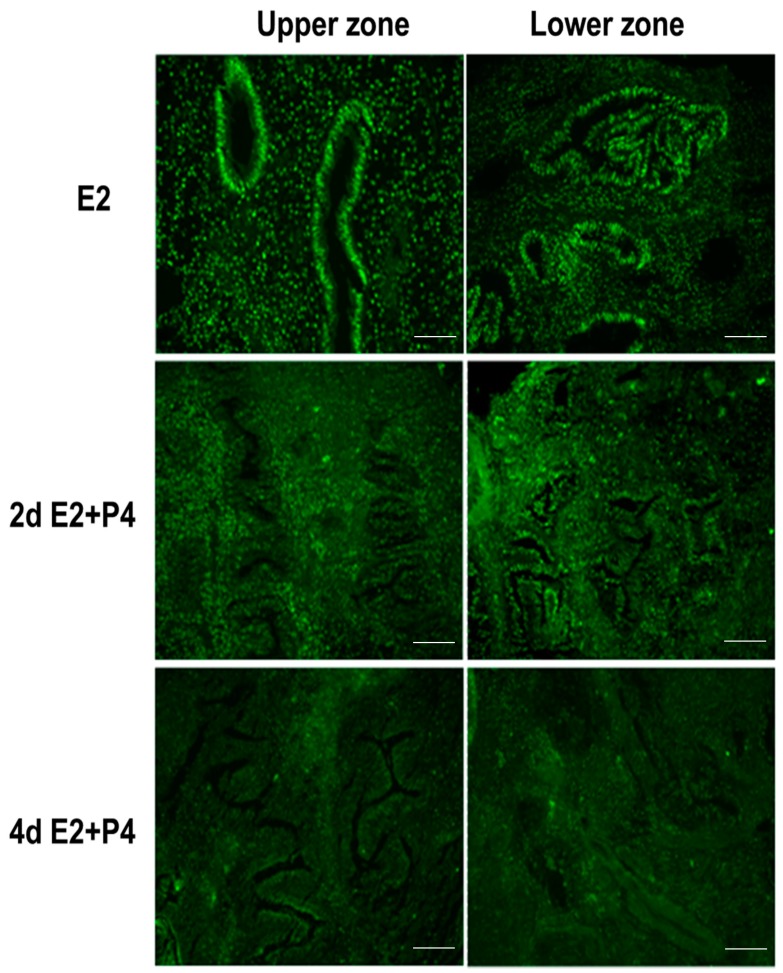
ER expression in the upper and lower zones of full thickness endometrial slices in culture. ER is markedly down-regulated, particularly in the upper zone glands after P4 treatment. Scale bar 200 µm; Original magnification 160×.

**Figure 6:**
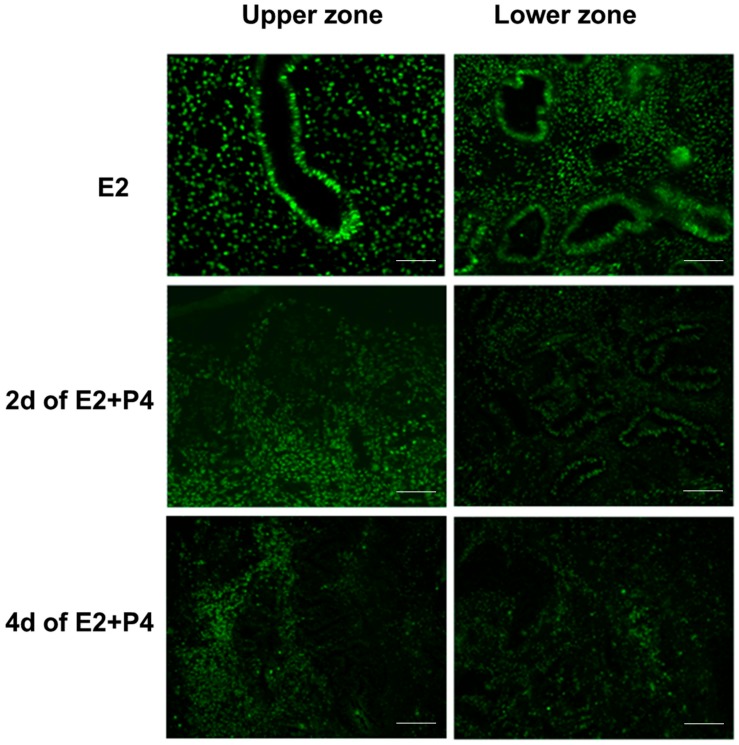
PR expression in the upper and lower zones of full thickness endometrial slices in culture. Compared to ER, more dramatic decrease in PR expression in both glands and stromal cells after P4 treatment. Scale bar 200 µm; Original magnification 160×.

It is well known that estrogen and progesterone regulate the endometrial estrogen receptor (ER) and progesterone receptor (PR) expression and cell proliferation depending on estrogen or progesterone dominance in the natural menstrual cycle. In an attempt to assess if the endometrial tissue slice culture can recapitulate the regulation of hormonal responses in a manner similar to the natural menstrual cycle, we examined the expression of ER, PR, and the nuclear proliferation marker Ki67 in response to E2 and P4. Tissue sections collected from the same block were used for the immunodetection of Ki67, ER, and PR. Consistent with the preservation of menstrual function in the endometrial 3D slice culture model, we observed that the cultures responded to E2 and P4 signaling by downregulating Ki67 (Figure 4), estrogen receptor (ER) (Figure 5), and progesterone receptor (PR) (Figure 6) expression in a manner similar to functional endometrium during menstrual cycle. 

### 3.3. Induction of Glandular Remodeling and Decidualization in Endometrial Tissue Air-Liquid Interface 3D Cultures

**Figure 7:**
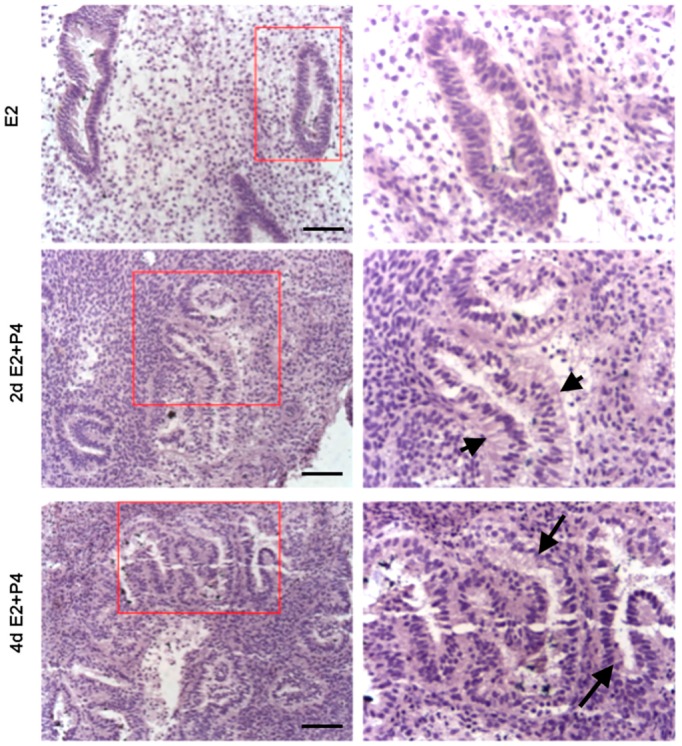
Histology of endometrial slices following E2 and different durations of E2 + P4 treatments. Arrow heads show subnuclear vacuolation in glands after treatment with 2 days of E2 + P4. Arrows show supranuclear vacuolation in glands after treatment with 4 days of E2 + P4. Scale bar 200 µm; Original magnification 160×.

**Figure 8:**
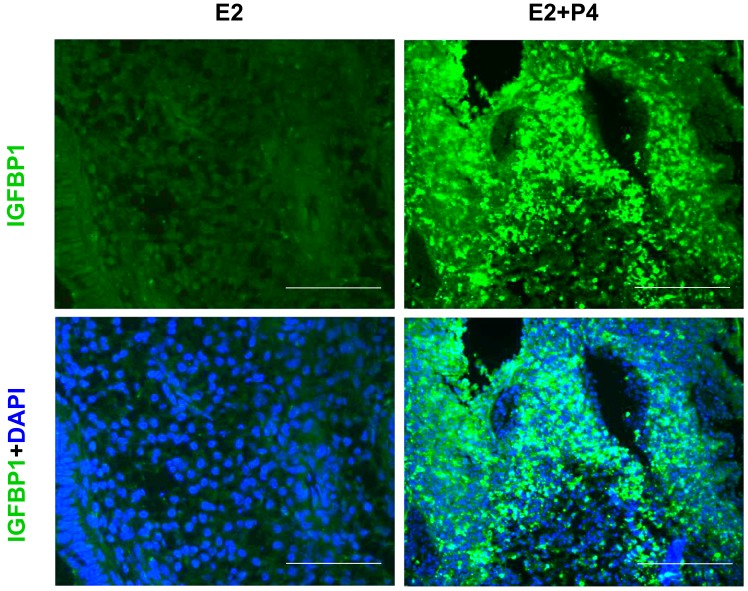
Decidual induction of Insulin-like Growth Factor Binding Protein 1 (IGFBP1) expression in the endometrial slices after the 21-day culture period. IGFBP1 immunofluorescence staining (green signals) in tissue sections derived from the endometrial slices in 3D culture treated with 4 days of E2 + P4. Nuclei were counterstained with 4′, 6-diamidino-2-phenylindole (DAPI). Scale bar 200 µm; Original magnification 160×.

The luminal and glandular epithelia of the endometrial tissue slices exhibited a simple columnar epithelium, a small glandular lumen and spindle-shaped stromal cells characteristic of an early proliferative phase prior to the P4 treatment (Figure 7). Following an E2 + P4 treatment regimen, histological examination of the slice culture revealed subnuclear vacuolation in glands, an expanded glandular lumen and glandular epithelial cells acquiring a low columnar phenotype. At the end of 4 days of E2 + P4, supranuclear vacuolation was evident in glands (Figure 7). These changes are typical of the morphological changes induced in the cyclic endometrium in vivo. In addition to these morphological changes in the glands, functional changes to stromal cells resembling a characteristic decidual morphology with enlargement were also evident. To further confirm if this represents a decidualization reaction, we assessed the expression of decidualization marker IGFBP1 in the tissue section derived from endometrial slices after the 21-day culture period. Consistent to an induction of decidualization, we observed an increased IGFBP1 expression in the 3D endometrial cultures following the E2 + P4 regimen (Figure 8). We did not use exogenous cyclic adenosine monophosphate (cAMP) to induce decidualization in order to assess the potential of the slice culture system as a physiologically relevant in vitro model that can decidualize with P4-dependent activation of endogenous cAMP pathway in a manner similar to the in vivo situations [51]. The observation that the decidualization can occur in the 3D culture model after the 21-day culture period clearly demonstrates the decidual viability after long-term culture and further encourages more detailed mechanistic studies on transcription factors critical for decidualization and to develop the model for studies on implantation and early pregnancy. 

Although recent studies have demonstrated that endometrial organoid cultures can be used to achieve the complexity of organ structures to a considerable degree through cellular self-assembly and can be used as preclinical models to study the physiology and pathology of human endometrium [17,18,19,20,21,22,23,24], these models do not fully recapitulate the regulatory mechanisms of endometrium in vivo. Herein with the use of full thickness endometrial tissue slice cultures we developed a unique model that dramatically enhances endometrial tissue viability and recapitulates the zone-specific changes seen in normal physiological conditions of endometrium [52,53,54]. It has been reported that the pattern of expression and localization of signaling molecules in cells at various zones of endometrium vary throughout the menstrual cycle but parallel to the cyclical changes in the endometrium; and aberrations in the expression pattern are associated with pathological conditions such as endometriosis [55,56,57]. Therefore, temporally coordinated actions of a variety of proteins expressed in cells at various zones of the endometrium is required to execute the sequential phases of endometrial tissue remodeling during the menstrual cycle. The influence of such factors on endometrial cellular functions in a timely manner is largely dependent on the preservation of cells in the different zones of the endometrium. It is encouraging to note that the present study with the development of 3D endometrial tissue slice culture using collagen gels by incorporating an air-liquid interface offers a platform to preserve endometrial zonation, tissue viability and responsiveness for long periods up to 21 days in culture along with abilities to remodel the tissue in response to hormones. 

It is generally believed that three-dimensional (3D) tissue culture models provide the opportunity to explore the cell-cell interactions occurring between different cell types of the endometrium producing phenotypes more closely related to in vivo situations than the 2D tissue culture models. In this context, considerable amount of success has been reported with the ex vivo culture of human endometrial tissue in 3D fibrin matrix that recapitulates several aspects of endometriosis [58,59,60,61]. However, these model systems largely involve the stromal and epithelial outgrowth from the endometrial tissue samples that limits its scope of being used as a tool mainly in endometriosis research [58,59,60,61]. Besides such models, magnetically bioprinted 3D in vitro models such as the uterine rings have been developed and used for the assessment of uterine contractility in physiological and pathological settings [62]. Although these models might serve as valuable tools for the study of uterine contractions, they cannot model the zone-specific changes in the cyclic endometrium since this system was designed to bioprint human myometrial cells into rings that lack the distinct zones of the endometrium. To study the zone-specific changes associated with physiological changes in the endometrium, attempts have been made previously to culture human endometrial biopsy specimens on dishes coated with matrigel or cell culture inserts [63,64,65]. However, these models suffered the major disadvantage of cell death that started within a short period of 8–24 h in the culture system [63,64] leading to the marked loss of tissue viability and responsiveness beyond five days [65] besides other disadvantages associated with loss of PR in the stroma [63]. Thus, our study has established that the 3D natural scaffold of type I collagen gel that include an air-liquid interface offers a platform for endometrial tissue slice culture with sustained tissue viability and responsiveness to steroid hormone-induced endometrial growth, structural remodeling, and turnover. 

## 4. Conclusions

Here, we have developed a 3D culture system with full thickness endometrial tissue slice that recapitulates the endometrial zone-specific changes in response to ovarian steroid hormones under tissue culture settings and can offer an alternative to accurately replicate the complex physiological properties of anatomical structures characteristic of an intact endometrium. Our results demonstrate that human endometrial tissue slices can be successfully cultured by incorporating an air–liquid interface into a 3D matrix scaffold of type I collagen gel, which allows sustained tissue viability over three weeks. This long-term slice culture method provides a unique in vivo-like microenvironment for studies of human endometrial repair, regeneration, and remodeling.
